# Short-Term Effects of Electroconvulsive Therapy on Subjective and Actigraphy-Assessed Sleep Parameters in Severely Depressed Inpatients

**DOI:** 10.1155/2015/764649

**Published:** 2015-01-06

**Authors:** Alexander Hoogerhoud, Andreia W. P. Hazewinkel, Robert H. A. M. Reijntjens, Irene M. van Vliet, Martijn S. van Noorden, Gert Jan Lammers, J. Gert van Dijk, Erik J. Giltay

**Affiliations:** ^1^Department of Psychiatry, Leiden University Medical Center, P.O. Box 9600, 2300 RC Leiden, Netherlands; ^2^Department of Neurology and Clinical Neurophysiology, Leiden University Medical Center, P.O. Box 9600, 2300 RC Leiden, Netherlands

## Abstract

*Background*. Sleep disturbances are a key feature of major depression. Electroconvulsive treatment (ECT) may improve polysomnography-assessed sleep characteristics, but its short-term effects on actigraphy-assessed and subjective sleep characteristics are unknown. We therefore aimed to assess the effects of ECT on subjective and objective sleep parameters in a proof-of-principle study. *Methods*. We assessed subjective and objective sleep parameters in 12 severely depressed patients up to 5 consecutive days during their ECT course, corresponding to a total of 43 nights (including 19 ECT sessions). The 12 patients were 83% female and on average 62 (standard deviation (SD) 14) years old and had an average MADRS score of 40 at baseline (SD 21). *Results*. Subjective and objective sleep parameters were not directly affected by ECT. The subjective sleep efficiency parameter was similar on the day after ECT and other days. ECT did not affect the number of errors in the Sustained Attention to Response Task. Patients subjectively underestimated their total sleep time by 1.4 hours (*P* < 0.001) compared to actigraphy-assessed sleep duration. *Conclusion*. ECT did not affect subjective and actigraphy-assessed sleep in the short term. Depressed patients profoundly underestimated their sleep duration.

## 1. Introduction

Sleep disturbances are a key feature found in patients with a major depressive disorder (MDD). Most patients suffer from difficulties with falling and staying asleep, early morning awakenings, and nonrestorative sleep [1]. Sleep disturbances are perceived as distressing and debilitating. These disturbances can lead to fatigue and social and occupational dysfunction [2] and are an important reason to seek medical attention [3]. The prevalence of DSM-IV primary insomnia in the general population is about 6% [4] versus 60–90% of patients diagnosed with MDD [5, 6], with the highest prevalence proportions found in inpatients [7].

On a short term, sleep loss and other sleep disturbances can cause a range of neurobehavioral deficits, including lapses of attention, slowed working memory, and reduced cognitive capacity [8, 9]. The strongest effects of sleep deprivation were reported in the domains of alertness and sustained attention [10]. Sleep disturbances may not only be a symptom of MDD but contribute to it as well, as supported by the following findings: (1) insomnia was associated with doubling the risk of developing MDD in a meta-analysis consisting of 21 longitudinal cohort studies [11] and sleep disturbances partially predicted MDD according to a meta-analysis of 23 studies on adolescent populations [12], (2) persisting insomnia increased the risks of suicidality, relapse, and recurrence of MDD [7, 13], and (3) the chance of remission increased when antidepressants were combined with treatment aimed at insomnia (e.g., sleep medication and cognitive behavioural therapy) [14].

A limited number of studies investigated effects of electroconvulsive treatment (ECT) on sleep disturbances. ECT is an effective treatment for severe MDD, with 50% remission and 80% response proportions. While the latency between sleep onset and the first episode of rapid eye movement (REM) sleep is typically shortened in depressed patients, small studies in 11 and 25 patients showed that ECT improved sleep parameters, decreased durations of REM sleep, and increased REM sleep latency as assessed using polysomnography [15, 16]. It might be hypothesized that one of the antidepressant mechanisms of ECT operates through its beneficial effect on sleep duration and quality, as control of the sleep/wake cycle involves numerous brain areas and pathways [17].

Our clinical impression was that some patients show marked improvements in subjective sleep even before the antidepressant effects of ECT became noticeable. We aimed to ascertain whether ECT had any short-term effect on subjective and objective sleep parameters by following 12 patients up to 5 consecutive days (4 nights) during which most received 2 ECT sessions. As a proof-of-principle study, wrist actigraphy was used to estimate sleep-wake schedules by measurement of activity. This is a noninvasive and objective method that allows for longitudinal evaluation of sleep in the setting of severely depressed inpatients [18].

## 2. Methods

### 2.1. Participants

**Figure 1:**
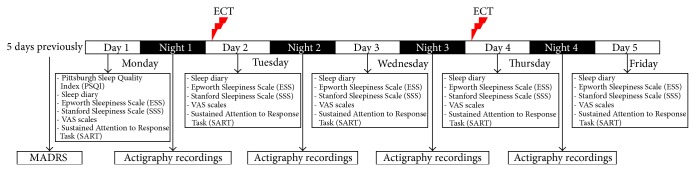
Schematic representation of the study protocol and flowchart.

Patients receiving index or maintenance ECT for a depressive episode were recruited from the Department of Psychiatry, Leiden University Medical Centre, Netherlands. Indications for ECT were depressive episodes during a major depressive, schizoaffective, or bipolar disorder (types I and II). Patients were eligible at any moment during their ECT course. We excluded patients suffering from motor disorders (e.g., Parkinson's disease, tremor, and severe akathisia) or disorders known to disrupt sleep. Antidepressant medications were generally phased out before the start of their present ECT course, but patients were allowed to use low-dose antipsychotics and sleep medication (i.e., benzodiazepines) at stable doses during the study. We aimed for continuous assessments from Monday through Friday, for example, 5 days and 4 nights. If this was unfeasible a minimum duration of 3 days and 2 nights with at least one ECT session was stipulated. ECT was administered every Tuesday and Thursday (Figure 1).

We included 12 out of consecutive 15 patients, assessed for a total of 43 nights; the other three declined participation. Of those nights, 19 directly followed the day of ECT. Eight patients (67%) were studied for the full five days and four nights, three were studied during three nights, and one was studied during two nights. During follow-up, 10 participants slept in the psychiatry ward and 2 patients on maintenance ECT slept at home. The research assistant visited each patient daily to repeat instructions, because of possible short-term memory loss due to ECT, and to guide them through all assessments. All patients followed a daytime schedule of routine activities in the psychiatry ward, except for the two patients on maintenance ECT. The study protocol (version 9.0) was approved by the Leiden University Medical Centre Ethics Committee, and all patients gave written informed consent.

### 2.2. Subjective Sleep and Other Parameters

Sleep diaries, questionnaires, and visual analog scales were filled in every morning to assess subjective sleep quality and mood-related symptoms. The daily sleep diary consisted of (1) time going to bed, (2) estimated duration from going to bed until falling asleep, (3) estimated number of awakenings after sleep onset, (4) time of final awakening in one rest period, and (5) time getting out of bed. Next to total sleep time calculated from sleep diaries, patients reported their (6) Estimated Total Sleep Time (ETST) during the previous night [19].

Daytime sleepiness and vigilance were assessed by two questionnaires. The Epworth Sleepiness Scale (ESS) is an 8-item questionnaire (Cronbach's alpha 0.88) [20] in which participants rate their likelihood of falling asleep during eight situations on a 0–3 Likert scale (0 = would never doze; 3 = high chance of dozing) [21]. The total score ranges from 0 to 24, with scores >10 being indicative of excessive daytime sleepiness [20, 21]. The Stanford Sleepiness Scale (SSS) is a 1-item Likert-type scale, with seven progressive levels of present state vigilance [22].

Every day during follow-up the participants were asked to complete 7 visual analogue scales (VAS that ranged from 0 to 10) for (1) pain, (2) previous' night sleep quality, (3) daytime fatigue, (4) overnight stress and rumination, (5) daytime sleepiness, (6) depressed mood, and (7) interest in activities.

### 2.3. Objective Sleep Parameters

The primary outcome measures were the objectively measured sleep parameters by actigraphy, which can accurately assess night-to-night variability of a given individual's sleep and is considered sensitive to treatment effects [23, 24]. Patients were instructed to wear the actigraph continuously throughout the study on their nondominant hand (except during ECT or showering). The actigraph is a piezoelectric accelerometer that measures the degree and intensity of motions during one-minute periods. In this study two versions of actigraph devices were used, both created by the same manufacturer (CamNtech Ltd., Cambridge, UK). The first 2 participants were supplied with version 5.0 and the following 10 were given version 8.0 (CamNtech Ltd., Cambridge, UK). A previously defined algorithm [25] (calibrated against the gold-standard polysomnography [23, 24]) was used to yield the following 10 sleep parameters: (1) time in bed; (2) sleep onset latency from time going to bed until actigraphically recorded sleep onset; (3) assumed sleep time (AST) from the time a subject fell asleep until the last awakening in the morning; (4) wakefulness after sleep onset (i.e., minutes spent awake after sleep onset); (5) actual wake as a percentage of total time in bed; (6) total sleep time (TST) calculated by subtracting time spent awake from assumed sleep time; (7) number of awakenings; (8) sleep efficiency as the percentage of total sleep time and time in bed; (9) mean activity score for each epoch of 60 sec during one rest period; and (10) fragmentation index (i.e., an indicator of sleep disruption composed of the relative number of wake bouts).

The Sustained Attention to Response Task (SART) was used to assess sustained attention, alertness, and concentration. Sustained attention is associated with daytime sleepiness and previous night sleep quality [26, 27]. Numbers ranging from 1 to 9 appear on a screen while the patient is asked to press the button every time a number appears on the screen and to inhibit this response when number three appears. The SART requires 4.3 minutes, during which period 225 numbers are shown in different fonts and sizes. Patients were asked to perform this test daily at approximately 14:00 hrs.

### 2.4. Other Parameters

Baseline characteristics consisted of sociodemographic parameters (i.e., sex and age), the preceding (last week's) Montgomery-Åsberg Depression Rating Scale (MADRS), and the Pittsburgh Sleep Quality Index (PSQI) [28]. The PSQI assesses previous' month sleep problems and has seven subscales (all scored 0–3): subjective sleep quality, sleep latency, sleep duration, habitual sleep efficiency, sleep disturbances, use of sleep medication, and daytime dysfunction. It has good reliability and high internal consistency, with Cronbach's alpha ranging from 0.83 to 0.46 for different subscales. A total score of ≥6 is indicative of significant sleep problems [28]. We also ascertained the number of ECT treatments previously received during the present ECT course, the DSM-IV axis I disorders diagnosed by the psychiatrist, and psychotropic medication use.

### 2.5. Statistical Analysis

Sociodemographic and baseline characteristics were summarized as means (+/− standard deviation (SD)) for continuous variable and as numbers (percentages) for categorical variables. Differences between subgroups were analysed with independent sample *t*-tests or chi-square analysis, where appropriate. The sleep diary of subject number 2 reported an estimated sleep duration of 0 hours during nights 2 and 4; we imputed the highest value available in our database for sleep onset latency (i.e., 290 minutes) and the lowest value in our dataset for sleep efficiency (i.e., 29%) for these 2 time points. Because data of this study were nested (repeated measurements within an individual) with some missing values on several days, multilevel regression analysis (linear mixed-models) was used to analyse the effects of a preceding ECT session on sleep-related parameters. A compound symmetry covariance structure was used consisting of up to 5 time points (i.e., lower level) and the patients (i.e., higher level). A multivariate model was constructed adjusting for age and sex. Next, multilevel regression analyses were also used to yield beta-coefficients for the associations between subjective and objective sleep parameters, taking into account that the up to 4 measurements within patients were not independent. All tests were two-tailed with *P* < 0.05 denoting statistical significance. The software used was SPSS version 20.0 (IBM Corporation, Armonk, NY).

## 3. Results

### 3.1. Participant Characteristics

Baseline characteristics of the studied population are shown in Table 1. The mean age was 61.9 ± (SD) 14.2 years and 83% (*n* = 10) were female. Ten patients were diagnosed with MDD, five with psychotic features, one with depression and bipolar disorder, and one with depression and schizoaffective disorder. When our measurements started, patients had received already an average of 10 preceding electroconvulsive sessions on average; four patients were on maintenance ECT. Sleep medication, mainly zolpidem, was used by 11 patients; two used antidepressants and four used antipsychotics. The MADRS score, measured five days before onset of the study, was 40.3 ± 21.0. Eleven (92%) patients met clinically significant criteria for poor sleep in the previous month (PSQI ≥ 6). The average PSQI score was 12 ± 4.6, indicating very poor sleep quality.

### 3.2. Subjective Parameters

Table 2 shows the direct effects of ECT on subjective parameters, covering either the first few hours after ECT or the effect on sleep during the night following ECT. Depressed mood and interest in activities VAS scores improved after ECT, compared to other days, which approached statistical significance. As some patients developed headache or body aches after ECT, pain scores were also higher on ECT days (*P* = 0.06). However, other subjective parameters and the mean VAS sleep quality were similar on nights following ECT compared to other nights (mean difference 0.53, SE 0.46, and *P* = 0.27).

### 3.3. Objective Parameters

Table 3 shows the effects of ECT on objective parameters. The SART results obtained on 20 days of ECT were compared to 28 days that patients did not receive ECT, and the actigraphy-assessed sleep parameters of 18 nights following ECT were compared to 24 other nights. There was no significant difference for sustained attention between the two groups for either false positive, false negative, total errors, or reaction latency. As patients were woken up earlier by nurses on mornings they received ECT, patients spent on average 0.90 less hours (SE 0.23, *P* = 0.001) less time in bed, and the mean AST and TST were, respectively, 0.74 hours (SE 0.24, *P* = 0.004) and 0.70 hours (SE 0.22, *P* = 0.003) shorter. Sleep efficiency was however not significantly different (mean difference 1.17, SE 1.37, and *P* = 0.40).

### 3.4. Comparing Objective with Subjective Sleep Parameters

**Figure 2:**
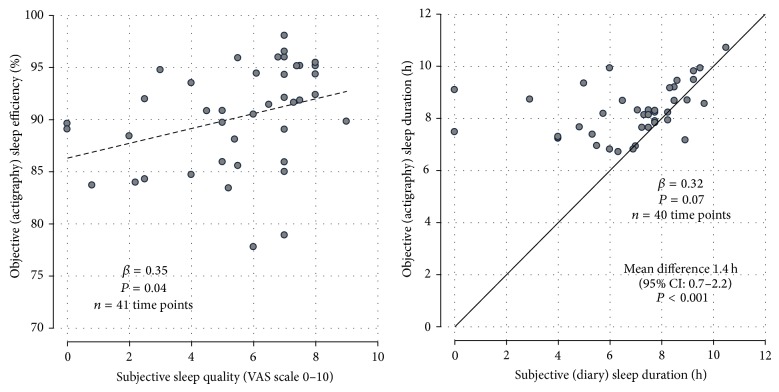
Scatterplots showing the relationship between subjective and objective sleep parameters.

Figure 2 shows that subjective sleep quality was weakly associated with actigraphy-assessed sleep efficiency (*β* = 0.35). Subjective sleep duration was also poorly associated with actigraphy-assessed sleep duration (*β* = 0.32). Patients systematically underestimated their duration of sleep by on average 1.4 hours. Leaving out the data of the two nights in which a patient had reported no sleep at all did not alter this; the mean difference remained significant (difference 1.1 h; 95% confidence interval: 0.6–1.6; *P* < 0.001).

### 3.5. Discussion

In this relatively small pilot study, using actigraphy to ascertain the potential acute effects of ECT on sleep parameters, we found no differences in sleep quality between nights following ECT and other nights, in neither subjective (e.g., sleep diary reports, VAS scores, and daytime sleepiness scales) nor objective parameters (e.g., actigraphy assessment and SART-test results). In our group of depressed patients, we found that the duration of sleep was subjectively underestimated by patients. As a proof-of-principal actigraphy, the SART and sleep questionnaires provide a pragmatic way to assess sleep in severely depressed patients who undergo ECT.

Our findings need to be interpreted in the light of some limitations. First, we included relatively few and mostly elderly patients, although measurements were performed on several days. This increased statistical power and yielded the opportunity to compare within-patient day-to-day changes. Although results need to be confirmed in younger study populations, we see no reason however why these methods would be more difficult to apply in younger patients. Second, our impression was that patients starting an ECT course were often too ill to comply to the study protocol, but future studies should explore whether ECT might have short-term effects on sleep particularly during the first few ECT sessions (at baseline). Thirdly, the total sleep time in depressed patients (of about 6.5 h) may be overestimated in patients who lie motionless in bed whilst awake. When no wrist activity is recorded, these awake epochs are scored as asleep [29]. However, actigraphy validly assessed sleep time compared to polysomnography in a study of depressed patients [30], and, as we measured on several nights, a systematic error would not have affected night-to-night variability. Fourth, as patients were woken up almost an hour earlier the days that ECT was scheduled, we only used the sleep indices that were corrected for sleep duration/time in bed to base our conclusions on. Moreover, anaesthetics may have influenced sleep quality in the nights following ECT. Fifth, actigraphy cannot assess sleep stages reliably and therefore cannot measure these subtler aspects of sleep. Polysomnography showed shorter REM-sleep latencies, higher percentages of REM sleep, and less slow wave sleep in depressed patients compared to healthy individuals [15, 16]. Finally, part of the study population used psychotropic medication (i.e., benzodiazepines, antidepressants, and antipsychotics), though at stable doses, which might have affected sleep and cognitive functioning.

We found that the accuracy of sleep diary reports was poor when we compared the results to the actigraphy recordings. Although this is a common observation in insomnia patients, this may also be due to cognitive side effects of ECT [31] and is consistent with previous findings. A group of 68 healthy college students, aged 18 to 28 years, reported going to bed on average one hour earlier than the time ascertained from actigraphy [32]. The systematic bias in sleep time reports can be interpreted as a reflection of the patient's perception of sleep duration and quality. We found a moderate association between subjective and actigraphy-assessed sleep duration, which corroborates with the somewhat higher correlation of 0.51 in a group of 39 depressed bipolar patients [33]. Yet, subjective sleep onset latency did not significantly correlate with actigraphy-assessed sleep onset latency in 52 depressed insomniacs but did correlate with the time spent awake after sleep onset and the total sleep time [34].

Although the exact mechanisms of action of ECT have not yet been elucidated several hypotheses about the working action exist. Among these hypotheses, the neuroendocrine theory, proposed by Fink and Ottosson [35], postulates that the antidepressant efficacy of convulsive therapy results from the persistent release of hypothalamic substances that mediate mood changes from depression to normal mood states with attendant modification in vegetative functions (e.g., improvement of disturbed sleep patterns). It is also hypothesized that ECT increases neurotransmission in general, normalizes neuroendocrine functioning, and increases neuronal outgrow and the number of synapses, resulting in improvement of brain function in general and cognitive functioning, sleep, and mood specifically [35, 36].

We conclude that ECT did not affect subjective and objective sleep parameters on the short term. Nevertheless, we showed in this proof-of-principle study that actigraphy, the SART, and questionnaires can be used to assess sleep parameters in patients undergoing ECT. Actigraphy adds value to subjective sleep parameters that seem to be distorted by depressive symptoms and ECT. Future studies should include more patients undergoing ECT and evaluate more nights or should include baseline measurements before the start of an ECT course and end after completion of the ECT course.

## Figures and Tables

**Table 1:** Baseline sociodemographic and clinical characteristics of 12 patients undergoing ECT.

	Number or mean (±SD)
Female sex, *n* (%)	10 (83%)
Age (year), mean (SD)	61.9 ± 14.2
Clinical diagnosis, *n* (%)	
(i) Unipolar major depressive disorder	10 (83%)
(ii) Bipolar I disorder, depressive episode	1 (8%)
(iii) Schizoaffective disorder	1 (8%)
Frequency of ECT treatment	
(i) 2/week, *n* (%)	8 (67%)
(ii) Maintenance ECT	4 (33%)
Number of previous ECT sessions during present ECT course, mean (SD)	9.9 ± 6.8
Concomitant psychotropics, *n* (%)	
(i) Zolpidem	9 (75%)
(ii) Benzodiazepines	6 (50%)
(iii) Antidepressants	2 (17%)
(iv) Antipsychotics	4 (33%)
Montgomery-Åsberg Depression Rating Scale (MADRS), mean (SD)	40.3 ± 21.0
Pittsburgh Sleep Quality Index (PSQI), mean (SD)	12 ± 4.6

**Table 2:** Effects of electroconvulsive therapy on subjective parameters in 12 patients with a depressive episode.

	*n*	ECT on present day	*n*	ECT not on present day	Test	*P* value
Epworth Sleepiness Scale (ESS)	21	2.46 (SE 1.45)	34	2.16 (SE 1.42)	*F*(1, 42.2) = 0.22	0.64
Stanford Sleepiness Scale (SSS)	21	3.12 (SE 0.39)	34	2.89 (SE 0.37)	*F*(1, 42.4) = 1.02	0.32
VAS scores						
(i) Depressed mood	21	4.63 (SE 1.19)	34	5.54 (SE 1.18)	*F*(1, 42.2) = 3.79	0.06
(ii) Interest in activities	21	3.92 (SE 0.83)	34	4.66 (SE 0.80)	*F*(1, 42.3) = 3.06	0.09
(iii) Pain	21	3.93 (SE 0.97)	34	2.96 (SE 0.94)	*F*(1, 42.2) = 3.78	0.06
(iv) Daytime sleepiness	21	2.33 (SE 1.01)	34	2.26 (SE 0.98)	*F*(1, 42.3) = 0.02	0.88
(v) Fatigue	21	4.53 (SE 0.80)	34	4.70 (SE 0.75)	*F*(1, 42.6) = 0.09	0.76

	*n*	ECT on previous day	*n*	ECT not on previous day	Test	*P* value

Time in bed (hours)	19	9.42 (SE 0.37)	34	9.23 (SE 0.38)	*F*(1, 40.8) = 0.53	0.47
Sleep onset latency (min)	19	46.8 (SE 19.3)	34	42.3 (SE 18.2)	*F*(1, 41.7) = 0.07	0.79
Sleep duration (hours) according to the following						
(i) Sleep diary	19	6.93 (SE 0.85)	34	7.15 (SE 0.83)	*F*(1, 40.4) = 0.30	0.58
(ii) Estimated sleep duration	19	6.52 (SE 0.71)	34	6.69 (SE 0.69)	*F*(1, 40.9) = 0.12	0.73
Sleep efficiency (%)	19	75.0 (SE 7.19)	34	78.0 (SE 7.07)	*F*(1, 40.3) = 0.76	0.38
Number of awakenings	19	1.34 (SE 0.49)	34	1.22 (SE 0.46)	*F*(1, 41.5) = 0.09	0.77
VAS scores						
(i) Night-time stress and rumination	19	3.79 (SE 1.08)	35	3.82 (SE 1.05)	*F*(1, 41.2) = 0.01	0.94
(ii) Sleep quality	19	5.26 (SE 0.74)	35	5.79 (SE 0.71)	*F*(1, 41.5) = 1.28	0.27

ECT denoted electroconvulsive therapy.

Data are adjusted mean and standard errors (SE) between brackets.

Mean and *P* values are analysed by mixed models and were adjusted for age and sex.

*n* indicates the number of time points (i.e., days) of the measurements.

**Table 3:** Effects of electroconvulsive therapy on objective parameters in 12 patients with a depressive episode.

	*n*	ECT on present day	*n*	ECT not on present day	Test	*P* value
SART total number of error	20	20.5 (SE 5.9)	28	20.6 (SE 5.7)	*F*(1, 35.4) = 0.00	0.99
SART false positive reaction	20	10.7 (SE 1.5)	28	11.2 (SE 1.5)	*F*(1, 36.2) = 0.29	0.59
SART false negative reaction	20	9.8 (SE 5.2)	28	9.4 (SE 5.1)	*F*(1, 35.4) = 0.02	0.88
SART mean reaction latency	20	352 (SE 26)	28	346 (SE 26)	*F*(1, 35.5) = 0.40	0.53

	*n*	ECT on previous day	*n*	ECT not on previous day	Test	*P* value

Sleep onset latency (min)	18	14.8 (SE 5.1)	24	8.6 (SE 4.9)	*F*(1, 30.8) = 1.54	0.22
Wakefulness after sleep onset (min)	18	37.1 (SE 6.4)	24	34.2 (SE 6.1)	*F*(1, 28.5) = 0.21	0.65
Actual wake (%)	18	6.43 (SE 1.16)	24	6.62 (SE 1.11)	*F*(1, 28.8) = 0.03	0.86
Number of awakenings (no.)	18	18.2 (SE 14.1)	24	15.9 (SE 2.1)	*F*(1, 29.5) = 1.07	0.31
Sleep efficiency (%)	18	90.7 (SE 1.5)	24	91.8 (SE 1.4)	*F*(1, 29.5) = 0.73	0.40
Activity score/60 sec	18	8.34 (SE 2.01)	24	9.78 (SE 1.99)	*F*(1, 30.1) = 0.39	0.54
Fragmentation index	18	24.4 (SE 3.5)	24	19.0 (SE 3.4)	*F*(1, 29.2) = 3.22	0.08

ECT denoted electroconvulsive therapy. SART denotes Sustained Attention to Response Task.

Data are adjusted mean and standard errors (SE) between brackets.

All means and *P* values were analysed by mixed models. Total number of errors, false positive reactions, and false negative reactions were adjusted for age, sex, and mean reaction latency, whereas the mean reaction latency was adjusted for age, sex, and standard deviation of the reaction latency. Actigraphy parameters were adjusted for age and sex.

*n* indicates the number of time points (i.e., days) of the measurements.
